# Virtual reality using games for improving physical functioning in older adults: a systematic review

**DOI:** 10.1186/1743-0003-11-156

**Published:** 2014-11-15

**Authors:** Karina Iglesia Molina, Natalia Aquaroni Ricci, Suzana Albuquerque de Moraes, Monica Rodrigues Perracini

**Affiliations:** Master’s and Doctoral Programs in Physical Therapy, Universidade Cidade de São Paulo - UNICID, Rua Cesáreo Galeno, 448, Tatuapé, Sp 03071-000 Brazil

**Keywords:** Virtual reality therapy, Video game, Physical exercise, Randomized controlled trial, Aged

## Abstract

The use of virtual reality through exergames or active video game, i.e. a new form of interactive gaming, as a complementary tool in rehabilitation has been a frequent focus in research and clinical practice in the last few years. However, evidence of their effectiveness is scarce in the older population. This review aim to provide a summary of the effects of exergames in improving physical functioning in older adults. A search for randomized controlled trials was performed in the databases EMBASE, MEDLINE, PsyInfo, Cochrane data base, PEDro and ISI Web of Knowledge. Results from the included studies were analyzed through a critical review and methodological quality by the PEDro scale. Thirteen studies were included in the review. The most common apparatus for exergames intervention was the Nintendo Wii gaming console (8 studies), followed by computers games, Dance video game with pad (two studies each) and only one study with the Balance Rehabilitation Unit. The Timed Up and Go was the most frequently used instrument to assess physical functioning (7 studies). According to the PEDro scale, most of the studies presented methodological problems, with a high proportion of scores below 5 points (8 studies). The exergames protocols and their duration varied widely, and the benefits for physical function in older people remain inconclusive. However, a consensus between studies is the positive motivational aspect that the use of exergames provides. Further studies are needed in order to achieve better methodological quality, external validity and provide stronger scientific evidence.

**Electronic supplementary material:**

The online version of this article (doi:10.1186/1743-0003-11-156) contains supplementary material, which is available to authorized users.

## Introduction

The use of virtual reality (VR) using interactive games, as a complementary tool in rehabilitation has been a frequent focus of research since the late 1990′s, with a considerable increase in the number of publications in the last few years [1]. VR makes it possible to practice activities within enriched, secure and challenging environments, thereby favoring motor learning and neural plasticity [1, 2]. In addition, it is considered to be a feasible strategy for intervention and an alternative complement to conventional exercises [3].

Exercise is well recognized as a beneficial intervention to enhance physical functioning in older adults, ultimately improving overall health [4]. Some studies suggest that exercise using VR in elderly patients promotes improvements in mobility [5, 6], in muscular strength of the lower limbs [7], in cognition, mainly of executive functions [6], in balance control [1, 8–11], in reaction time [5] and also helps to prevent falls [1, 12, 13]. Although these studies might be compelling, there is not enough evidence to support that exercises using VR would promote better or even similar improvements in physical functioning, when compared with regular exercise programs[14].

Older people present diminished mobility and balance control as a result of morphological and functional changes in the central and peripheral nervous system related to age [15], to the presence of illnesses and to disuse. In order to overcome these negative changes, challenging, intensive and repetitive motor training through specific therapeutic exercises, such as those involving multi-segment coordination, anticipatory postural adjustments and tasks with divided attention can be used to enhance neuroplasticity [15, 16]. However the use of exergaming could optimize motor learning by combining physical and cognitive demands in an attractive and interactive way, motivating players to focus their attention not on the movements itself, but on the outcome of the movements in the game [2, 17].

There is a greater interest in the use of VR for therapeutic purposes [14, 17–25]. Systematic reviews concerning VR have been carried out in populations with specific health conditions, such as post-stroke patients and those with balance disturbances or falls [18, 20, 22–24], within different therapeutic settings [21] and in samples covering a range of age groups [14, 17]. Minor or inconclusive positive improvements have been demonstrated with the use of VR in individual studies. However, systematic reviews reveal notable lack of significant methodological quality studies and reinforce that there is insufficient information to recommend interventions with exercise using VR [14, 21]. Particularly, the evidence is scarce regarding the efficacy and effectiveness of using VR through games, on an isolated basis or in addition to conventional motor therapies in the older population. Thus, the aim of this systematic review is to investigate the effectiveness of exercises using interactive games (exergames) in improving physical functioning in older adults.

## Review

### Method

#### Electronic search

To verify the range of publications on interventions through exercises using VR games in older people, searches were made on the following data bases: EMBASE, MEDLINE, PsyInfo, Cochrane *data base*, PEDro and ISI Web of Knowledge. Four thematic blocks were used with a series of synonyms and variants related to 1) virtual reality with games, 2) exercises/physiotherapy, 3) aged and 4) randomized clinical trial. The words in a single block were combined among themselves with the use of the OR boolean operator and the interaction between blocks with the AND operator, in accordance with the example: ((user-computer interface) OR (computers or microcomputers or computer systems or software) OR (computer simulation or computer-assisted instruction or therapy computer-assisted) OR (computer graphics or video games or touch) OR (virtual reality* or virtual-reality* or VR) OR (computer game* or computer interact*) OR (computer assist* therap* or computer assist* treat*) OR (computer generat* environment* or computer generat* object) OR (video game*) OR (haptics or haptic device*) OR (user computer interface)) AND ((exercise) OR (exercise movement techniques) OR (exercise therapy) OR (biofeedback) OR (exercis* or training or biofeedback ) OR (physical fitness) OR (exercise tolerance) OR (sports) OR (physical endurance) OR (exertion*) OR (exercis*) OR (sport*) OR (physical fitness or motion fitness or physical therap*) OR (physical* endur*) OR (strength* or isometric* or isotonic* or isokinetic* or aerobic* or endurance or weight*) OR (train*) OR (physical therapy modalities) OR (physiotherap*) OR (kinesiotherap*) OR (rehabilitation or rehab*) OR (resistance training) OR (exercis* or training)) AND ((older adult or older people or older person* or elderly or seniors or geriatric or frail or aged)) AND ((randomized controlled trial*) or (controlled clinical trial) or (random allocation) or (double-blind method) or (single-blind method) or (clinical trial) or (placebo) or (random*) or (blind*)).

The search was conducted from July to October 2013 being limited to scientific articles and with no restrictions regarding language and publication year. In addition, a manual search was carried out to complement the strategy of looking up research on the topic.

### Study selection

This review included randomized controlled clinical trials with a sample of older adults (≥60 years old) and main intervention of VR with physical exercises by way of interactive games that aimed at rehabilitation. The VR exergames treatment could be on an isolated basis or combined with other interventions. For comparison, the control could be either with no intervention or conventional treatment (physical exercises, functional training or education). To be considered a game was necessary to fill out at least some of the following characteristics: interactivity, entertainment, rules, presence of an opponent or the objective to win points, and the possibility of winning or losing.

Studies were excluded if they had only cognitive outcomes, samples composed of participants with specific clinical conditions (i.e. stroke, Parkinson, dementias, diabetes mellitus, vestibulopathy), game performed in sit position, VR just provides visual information and insufficient description of statistical analysis.

For the selection of studies, the publications resulting from the searches had their titles and abstracts analyzed by two independent assessors. The articles selected for reading were once again analyzed in terms of criteria for eligibility. In the case of discordances, in any phase of the selection process, a third reviewer was called in for a consensus analysis. The studies included for the final review were analyzed in terms of methodology with the extraction of data regarding the sample composition, interventions and outcomes description, and the effects of the interventions.

### Outcomes

The primary outcomes considered were mobility-related physical functioning measures. Emphasis was placed on measurements for physical mobility and body balance through objective and/or subjective tests and instruments that are easily interpreted and used in clinical practice. Secondary outcomes included measurements relating to body structure and function, such as laboratorial posture control (ie. postural sway test and dynamic posturography), muscle strength (ie. maximal isometric muscle force and grip strength), reaction time and laboratory gait measurement.

### Methodological analysis and quality

The methodological quality of the studies was assessed using the PEDro scale. This scale analyses 11 items for the interpretation of research relating to the internal and external validity of clinical trials in physiotherapy [26]. The PEDro total score varies from zero to 10, with no score for the first item and the higher the score the better the methodological quality of the clinical trial. The ranking of the studies is available in the PEDro database [27]. In the absence of this ranking at PEDro database two assessors conducted the ranking on an independent basis and if there was disagreement, a third assessor was called in to reach a consensus.

Due to the variability between the interventions proposed and small study sample sizes it was not possible to conduct a metanalysis. A critical review analysis of the contents was performed.

## Results

**Figure 1:**
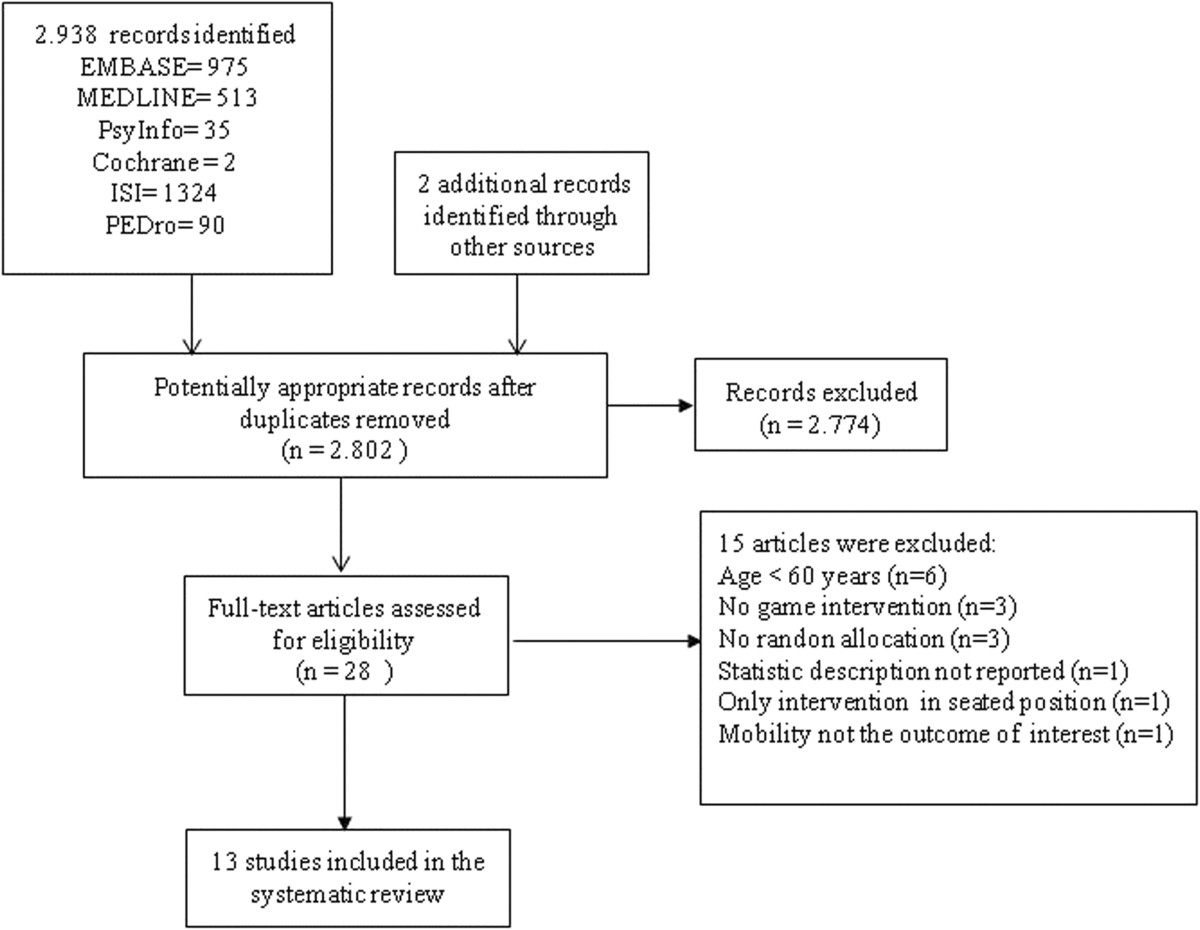
**Flowchart of literature search.**

As shown in Figure 1, a total of 2,940 references were found via the data base and manual searches. After the removal of duplicated references, the titles and abstracts were analyzed for 2,802 studies, with 28 articles being pre-selected for full reading. Of these, 15 were excluded because they failed to fit the inclusion criteria for this review, resulting in 13 articles for critical analysis of content. The synopsis of the articles analyzed can be found in Tables 1 and 2, in accordance with the type of device used (Wii console or others). The great majority of these articles (n = 11) [6–10, 12, 13, 28–31] was published in the last 2 years.

**Table 1:** **Data summary of randomized clinical trials using exergames with the Nintendo Wii gaming console**

Study	Sample	Trial Desing	Outcomes	Intervention	Effects Observed
**Bieryla & Dold**[9]	Healthy older adults from a local senior living community.	Randomized controlled trial with one month follow-up.	1) BBS.	**EG:** Wii Balance Board with Wii Fit.	- BBS scores significantly increased for EG participants. Post hoc analysis indicated a significant increase from pre-intervention to 1 month post-intervention but not a significant increase from pre-intervention to 1 week post-intervention. BBS score did not significantly change in the CG.
	N = 12		2) FAB.	The intervention consisted of a series of exercises and activities chosen from the yoga (half moon, chair, warrior), aerobic (torso twists), and balance games (soccer heading, ski jump) modes.	- There was no significant improvement in FAB, FRT and TUG for either group.
	81.5 ± 5.5 yrs		3) FRT.	Individual sessions 3x/week (30 min each session) for 3 weeks.	
	EG: n = 6 (withdraw = 1 during treatment and 1at follow-up)		4) TUG.	**CG:** No intervention.	
	CG: n = 6 (withdraw = 1)				
**Franco et al.**[28]	Community dwelling elders at low-income senior housing facility.	Randomized controlled trial.	1) BBS.	**EG1**: Wii Fit.	- Balance tests (BBS and Tinetti) improved in all 3 groups after the intervention period. There was a significant main effect of time (pre- to post-intervention) but no interaction between time (pre and post) and groups (Wii Fit, MOB, Control).
	N = 32		2) Tinetti-POMA	5 Wii Fit balance training games (soccer heading, ski jumping- slalom, tightrope, table tilt and balance bubble) with supplemental home exercises (balance and flexibility- daily).	
	78.27 *±* 6 yrs		3) SF-36.	- Individual sessions 2x/week (10-15 min each session) for 3 weeks.	
	EG1: n = 11		4) Wii Fit Enjoyment Questionnaire.	**EG2:** Matter of Balance program (MOB). The program uses a cognitive restructuring for coping strategies, strength training exercises (elastic resistance band) for strength and balance tasks to reduce fall risk.	- The groups were different at pre-test in SF-36 scores. There was no significant time change in SF-36 scores and non-significant group-time interaction.
	EG2: n = 11			Group sessions 2x/week (30-45 min each session) for 3 weeks.	- 81% of the EG1 participants reported high levels of enjoyment while playing the Wii games.
	CG: n = 10			**CG:** No intervention.	
**Jorgensen et al.**[7]	Community-dwelling older adults.	Randomized controlled trial.	1) Maximal voluntary contraction (MVC) of leg extensors.	**EG**: Nintendo Wii training.	- Between-group difference (pre-to-post changes) favoring the EG were evident in the MVC, RFD, TUG, FES-I, and Chair Stand Test. The CoP-VM did not differ between groups.
	N = 58		2) Postural balance- center of pressure velocity moment (CoP-VM).	Each training session was designed to include balance exercise games followed by a muscle exercise sequence. The participants could choose freely between 5 balance games (table tilt, slalom ski, perfect 10, tight rope tension, penguin slide), whereas a single exercise (standing rowing squat) was used for muscle conditioning.	- EG participants either agreed or strongly agreed with the statement that Wii training was fun and motivating.
	75 ± 6 yrs		3) Rapid force capacity (RFD).	- Individual sessions 2x/week (35 to 40 min each session) for 10 weeks	
	EG: n = 28 (withdraw = 7)		4) TUG.	**CG**: EVA insoles as placebo.	
	CG: n = 30 (withdraw = 2)		5) FES-I.	The participants in CG were instructed to wear EVA insoles in their shoes everyday for the entire duration of the trial. They received phone calls to check that problems with the EVA insoles had not emerged.	
			6) 30-sec repeated Chair Stand Test.	.	
			7) Likert scale regarding motivation toward Wii training.		
**Laver et al.**[29]	Geriatric Hospital	Feasibility Randomized controlled trial.	1) TUG.	**EG:** Nintendo Wii training.	- There was no difference between groups on univariable analyses for any measures.
	Rehabilitation		2) SPPB.	Treatment focused on balance tasks (weight shift on the balance board), strength exercises for the lower limb (sustained squats or single leg extension) and aerobic capacity (stepping on and off the balance board or walking on the spot).	Multivariable analyses (based on the number of intervention sessions) adjusting for length of stay, age, gender and baseline FIM showed that EG improved more on TUG and MBBS.
	Unit.		3) Modified BBS.	Individual sessions 5x/week (25 min each session) for the duration of the participant’s stay on the unit.	- No statistically significant differences were found between groups for the SPPB, Timed IADL Test, ABC Scale or EQ5D.
	N = 44		4) TIADL.	**CG**: Conventional physiotherapy.	- In either groups, participants reported some discomfort, being musculoskeletal pain the most frequent one. Within the CG, 1 serious adverse event (conscious collapse- vasovagal). Three participants from the EG fell while on the unit in comparison to one fall reported from CG.
	84.9 ± 4.5 yrs		5) FIM.	Treatment sessions included walk, transfers practice, walk up and down steps, balance tasks (standing on a foam block, tapping a balloon or reaching for objects), strength (e.g. use of light weights or stretches), aerobic and flexibility exercises.	
	EG: n = 22 (withdraw = 2)		6) ABC Scale.	- Individual sessions 5x/week (25 min each session) for the duration of the participant’s stay on the unit.	
	CG: n = 22		7) EQ5D.		
			8) Participant reports of discomfort and adverse events.		
**Maillot & Perrot**[6]	Independent older adults.	Randomized controlled trial.	1) Physical Measures:	**EG**: Nintendo Wii training.	- Follow-up tests showed greater improvement in the EG than in the CG for all the physical measures except the Back-Scratch upper-right and the Borg Scale ratings.
	N = 32		- chair-stand test;	Each session began with a warm-up and finished with a cool-down. The main session comprises games from the Wii Sports, Wii Fit, and Mario & Sonic on Olympic Games. The participants’ pairs change at each session with the intention of making the exergame playing more enjoyable and motivating adherence to the regimen.	- The comparisons for measures of executive function and processing speed show that improvement was significantly greater in the EG than in the CG.
	65 to 78 yrs		- arm curl test;	Pairs sessions 2x/week (30 min each session) for 12 weeks.	- For measures of visuospatial function, the difference between the EG and CG was not significant.
	EG: n = 16 (withdraw = 1)		- 6 MW (meters, borg scale max and mean HR);	**CG**: No intervention.	- 80% of EG participants agreed that the exergame training was manageable for seniors. All participants reported that they would like to continue with exergame activity, however only 40% considered acquiring a game console.
	CG: n = 16 (withdraw = 1)		- chair-sit-and-reach test;		
			- back-scratch test;		
			- 8 ft UG.		
			2) Cognitive battery:		
			- executive control tasks (Trail- Making test, Stroop Color Word Interference test, Letter Sets test, Matrix Reasoning test2, Digit Symbol Substitution test);		
			- visuospatial tasks (Spatial Span test, Directional Headings test, Mental Rotation test)		
			- processing-speed tasks subdivided into two categories: perceptual speed (Cancellation test and Number Comparison test) and psychomotor speed (the Reaction Time test and Plate Tapping test).		
			3) Impression questionnaire of the exergame program.		
**Pluchino et al.**[10]	Independent seniors.	Randomized controlled trial- pilot study.	1) TUG.	**EG 1:** Standardized Balance Exercise. Program consisted of 14 functional activities with a pronounced demand for balance (stepping on a compliant surface, walking forward 10 steps and pivoting 180°, alternately moving a weight between a high and low shelf situated just beyond 1 arm’s length and more).	- Significant increase in COP area across time (pre to post-test) was seen for the three groups. Differences were detected for the COP anterior-posterior excursion (max/ min/ SD) and velocity (max/min); and COP medial-lateral excursion (min) and velocity (max). No significant group X time interactions were detected for any COP measurements.
	N = 40		2) OLS.	**EG 2:** Tai Chi program.	- For dynamic posturography, significant improvements in the overall score (dynamic movement analysis score), and in 2 of the 3 linear and angular measures were seen for the sample.
	72.5 ± 8.40 yrs		3) FRT.	The program was based on the Tai Chi Sun-style. The program consisted of 12 movements using small forward and backward steps, as well as weight transfers from one leg to the other. The form also focused on posture alignment, slight bending of the knees and moving slowly with a gentle resistance.	- No significant differences were seen on time or group (EG1, EG2, EG3) X time (pre-post) interaction for any field test or questionnaire.
	EG1: n = 14 (withdraw = 6)		4) Tinetti- POMA.	**EG 3**: Wii Fit Balance Program.	
	EG2: n = 14 (withdraw = 3)		5) Postural Sway Test (force plate): COP area and velocity for medial-lateral/ anterior-posterior directions.	Games used for the balance program were: soccer heading, ski slalom, ski jump, table tilt, tightrope walk, river bubble, penguin slide, snowboard slalom and lotus focus. The games were based on the control of an on-screen avatar using body movements that are detected by the balance board. The training starts with participants playing each game for 7 min. During the second day they played 5 out of the 8 games for 10 min each. The 5 games played were chosen as follows: the 3 games in which they scored the lowest on the first day of training, and 2 games of their choice. For the duration of the study, the participants played 5 out of the 8 games per session for 10 minutes each. They first played the 3 games that were not played during the previous session. These were then followed by the 2 games in which they produced their lowest cumulative scores.	
	EG3: n = 12 (withdraw = 4)		6) Dynamic posturography	**EG1, 2, 3:** 5 min warm-up, 50 min of specific training, and 5 min of cool-down.	
			- DMA (dynamic motion analysis)	2x/ week (60 minutes each session) for 8 weeks.	
			- up and down, side to side, and anterior/posterior;		
			- translational and rotational movements including flexion/extension, lateral flexion, and core rotation.		
			7) FROP-Com.		
			8) FES.		
**Rendon et al.**[8]	Outpatient geriatric orthopaedic and balance physical therapy clinic	Randomized controlled trial.	1) 8 ft UG.	**EG:** Wii Fit balance games.	- Post-intervention measurements showed significant improvements for the EG in the 8 ft UG and ABC scale when compared with CG.
	N = 40		2) ABC scale.	Treatment comprises 8 min warm-up (stationary bicycle), 3 different balance games (lunges, single leg extensions and twists) and 8-min cool-down. Participants alternated the exercise game sequence week-to-week.	- Both groups scored in the ‘normal’ classification of depression scoring by the GDS (0–9 = normal). No significant between groups differences were seen for GDS.
	60 to 95 yrs		3) GDS.	Individual sessions 3×/week (35-45 min each session) for 6 weeks.	
	EG: n = 20 (withdraw = 4)			**CG:** No intervention.	
	CG: n = 20 (withdraw = 2)				
**Toulotte et al.**[31]	Healthy elderly living independently.	Randomized controlled trial.	1) OLS with EO/ EC.	**EG1:** Adapted Physical Activities. Participants undertook exercises to increase step length, step height, the mobility of the cervical rachis and ocular mobility in order to develop muscular strength, proprioception, flexibility, static balance with EO/ EC and dynamic balance. The difficulty of the exercises was increased at each session.	- After training subjects in EG1 and EG3 improved significantly in the Tinetti- POMA test and the scores of EG2 improved significantly only in the static part of the test.
	N = 36		2) Tinetti- POMA.	**EG2**: Wii Fit training.	- The number of times the suspended foot touched the floor during the OLS in EO/EC conditions decreased significantly after training for EG1 and EG3.
	75.09 ± 10.26 yrs		3) Wii Fit tests (center of gravity).	The participants used the Nintendo standardized video games (heading soccer, ski jumping, yoga, downhill skiing, game balls and tightrope walker). The training was personalized because the progress in training was based on the different levels into each video game.	- The percentage difference between right and left (center of gravity position) was significantly modified for EG3 and EG2, but no significant difference appeared in EG1 after treatment.
	EG1: n = 9			**EG3:** Adapted Physical Activities (30 min) + Wii Fit Training (30 min).	- There was no significant difference between pre and post-tests for the CG.
	EG2: n = 9			Training for EG3 were the same, but the number of repetitions was lower than the EG1 and EG 2.	
	EG3: n = 9			**EG1/EG2/EG3:** All subjects trained 1x/week (1 hour each session) for 20 weeks.	
	CG: n = 9			**CG:** The subjects watched television, played board games but no physical training was done.	

6 MW = 6 minute walk test; 8 ft UG = 8 foot Up and Go; ABC Scale = The Activities-specific Balance Confidence *Scale;* BBS = Berg Balance Scale; CG = Control Group; COP = Center of Pressure; DMA = Dynamic Motion Analysis; EG = Experimental Group; EQ5D = EuroQOL Five Dimensions Questionnaire; FAB = Fullerton Advanced Balance; FES-I = Falls Efficacy Scale International; FIM = Functional Independence Measure; FROP-Com = Falls Risk for Older People-Community Setting; FRT = *Functional Reach Test;* GDS = Geriatric Depression Scale; MBBS = Modified Berg Balance Scale; OLS = One Leg Stance SF-36 = SF-36 health survey; SPPB = Short Physical Performance Battery; TUG = Timed Up and Go; TIADL = Timed Instrumental Activities of Daily Living; Tinetti-POMA = *Tinetti* Performance Oriented Mobility Assessment.

**Table 2:** **Data summary of randomized clinical trials using exergames with other devices**

Study	Sample	Trial Desing	Outcomes	Intervention	Effects Observed
**Duque et al.**[12]	Community-dwelling elderly whit history of falls from the Falls and Fractures Clinic.	Randomized controlled trial.	1) Posturography (BRU) at six different conditions:	**EG:** Balance Rehabilitation Unit (BRU).	- After 6 weeks of intervention the EG showed significant increase in LOS and smaller elliptical areas of the EC on hard surface/ foam, optokinetic stimuli, and vertical/ horizontal visual-vestibular condition.
	N = 60		- LOS;	Treatment is consisted of visual-vestibular rehabilitation while standing and postural training virtual reality games (maze, breakfast and surfing) with increasing levels of complexity as the individual reported higher confidence and demonstrated learning of the correct postural control techniques required to pass to a higher level (maximum of 15 levels).	- After 9 months, as compared with the CG, the EG showed significantly higher level of LOS, and significantly smaller COP areas in the optokinetic stimuli and both vertical/ horizontal visual-vestibular condition. Elliptical areas of the EC on hard surface/ foam returned to the baseline values for EG.
	EG: n = 30 (withdraw = 2)		- COP EO/ EC on hard surface;	- Individual sessions 2x/week (at least 30 min each session) for 6 weeks. After 6 weeks participants received the usual care until complete 9 months.	- EG subjects reported a significantly lower number of falls and lower SAFFE score as compared with the CG.
	79.3 ± 10 yrs		- COP EC on foam;	**CG:** Usual care.	
	CG: n = 30 75 ± 8 yrs		- COP optokinetic stimuli;	All participants were given general recommendations and an evidence-based care plan on falls prevention.	
			- COP horizontal/ vertical visual-vestibular condition;	- 9 months.	
			2) Fall history.		
			3) Gait pattern (GAIT Rite® instrumented walkway): velocity, cadence, stride length and double support time.		
			4) Grip strength using a hand dynamometer.		
			5) Venous blood.		
			6) GDS.		
			7) SAFFE.		
**Schoene et al.**[13]	Residents at independent-living units.	Randomized controlled trial- pilot study.	1) CSRT using a step pad: reaction time, movement time and total response time.	**EG:** A computer unit and step pad at home.	
	N = 37		2) PPA:	The game required participants to synchronize their stepping with instructions presented on the screen. For each step, score and feedback was given in the center of the screen (perfect, good, miss). To introduce an additional cognitive load, a ‘bomb’, was randomly presented. If participants failed to avoid it the bomb ‘exploded’ as an indication of the error and points were correspondingly deducted from their game score. Participants were instructed how to use the system and play the stepping game in one (90 min) session in their homes and received a manual.	- Compared to the CG, the EG significantly improved their CSRT, PPA composite scores, as well as the postural sway and contrast sensitivity PPA sub-component scores. In addition, the EG improved significantly in the TUG dual-task.
	78 ± 5 yrs		- contrast sensitivity, proprioception of the lower extremities (knee joint position sense);	- Home individual sessions 2–3x/week (15–20 min each session) for 8 weeks.	- There were no differences between groups for any of the other outcome measures.
	EG: n = 18 (withdraw = 3)		- lower extremity strength (isometric knee extension);	**CG**: No intervention.	- EG participants played a median of 2.7 sessions/week and no adverse events were reported.
	CG: n = 19 (withdraw = 2)		- standing balance (postural sway on a compliant surface);		
			- simple hand reaction time.		
			3) TUG and TUG dual task.		
			4) 5STS.		
			5) AST.		
			6) TMT.		
			7) INHIB.		
			8) FES.		
**Pichierri et al.** **[**[30]	Participants from hostels for the aged.	Randomized controlled trial.	1) Foot placement test:	**EG:** Dance video game with pad.	- On foot placement performance, within-group comparison resulted in a significant improvement in ML deviation and walking velocity condition 2 in EG and no changes in the CG. Between-group comparisons revealed significant differences walking velocity condition 2 in favor of EG.
	N = 31		Condition 1: self-selected pace and place the right foot into target 1 (T1)/ Condition 2: place the right foot into target 1 and the left foot into target 2 (T2)/ Condition 3: step over an obstacle lying between the two targets.	EG received the CG program and in addition they performed a progressive video game dancing intervention.	- The within-group comparison revealed significant walking performance improvements throughout all the walking conditions for the EG. In contrast, in the CG improvements in walking performance were only observable for the normal and normal cognitive conditions. Significant between-group differences where observed in the fast cognitive condition. The EG showed a significant increase in walking speed and a decrease in single support time compared to the CG. Significant between-group differences for DTC were observed for the parameter single support time for both normal and fast walking speed favoring EG.
	86.2 ± 4.6 yrs		- M-L/ A-P deviation;	The dance video game was projected on a white wall and performed on metal dance pads. A scrolling display of arrows moving upwards across the screen cued each move, and the participants were asked to execute the indicated steps (forward, backward, right, or left) when the arrows reached the fixed raster graphic at the top of the screen, and in time with different songs. As the levels increased additional distracting visual cues, e.g., “bombs,” were presented and participants had to ignore these cues and focused on the arrows.	- FES-I questionnaire showed a reduction of concerns about falling in both groups after treatment. Between-group comparison resulted in no significant differences.
	EG: n = 15 (withdraw = 4)		- walking velocity condition 2 and 3;	Group sessions 2x/ week (40 min each physical program session) and in addition individual video game sessions (10-15 min) training for 12 weeks.	
	CG: n = 16 (withdraw = 5)		- M-L/ A-P contact with leading foot;	**CG**: Physical exercise program with progressive resistance and postural balance. The training session consisted of a warm-up (5 min), resistance training (25 min), and balance exercises (10 min).	
			- contact with subsequent foot;	Group sessions 2x/ week (40 min each session) training for 12 weeks.	
			- wrong foot.		
			2) Gait analysis: (GAITRiteW Platinum Version 4.0 software and the electronic walkway)		
			- Normal/ Fast/ Normal cognitive/ Fast cognitive: velocity, cadence, step time, cycle time, stance time, single/double support time, step length;		
			- dual task costs (DTC) of walking: percentage of loss relative to the single task walking performance (normal and fast walking).		
			3) Gaze behavior		
			4) FES-I.		
**Szturm et al.**[11]	Community-dwelling older adults attending at day hospital.	Randomized controlled trial.	1) BBS.	**EG:** Program coupled to computer games.	- Significant within-group and between group improvements in BBS scores were observed. Significant reductions in LOB counts on the foam surface and ABC scores were observed for the EG, but not for the CG.
	N = 30		2) TUG.	Participants received a program of dynamic balance exercises coupled with video game play, using a center-of-pressure position signal as the computer mouse. The tasks were performed while standing on a fixed floor surface, with progression to compliant foam. Three games were developed for use (under pressure, memory match and balloon burst)	- The EG exhibited significantly greater improvements in change scores for the BBS, ABC, LOB than the CG.
	EG: n = 14 (withdraw = 1)		3) Spatial-temporal gait pattern (gait speed, swing time, stance duration, double support, single support times, step length and step width).	Individual sessions 2x/ week (45 min each session) for 4 weeks.	- No significant within-group or between-group effect on spatiotemporal gait parameters and for the composite LOB on normal surface.
	80.5 ± 6 yrs			**CG:** Typical rehabilitation program at day hospital.	- At baseline there was a significant group difference in TUG time, with a worse time among the EG. Although improvements in TUG time did occur in both groups, the differences between groups were not significant.
	CG: n = 13 (withdraw = 2)		4) ABC scale.	Thera-Band and leg weights were used for strength exercises, and a cycle ergometer was used for endurance exercise. Balance exercises included hip flexion, side-leg raises, squats, and standing up from a chair and sitting down in the chair without using hands. Along with these exercises, there was an assessment of walking aids and a gait re-education program. Participants also were involved in an unsupervised walking program.	
	81 ± 7 yrs		5) MCTSIB - composite loss of balance (LOB) count for stability tasks performed on a fixed floor and on a compliant surface.	Individual sessions 2x/ week, (45 min each session) for 4 weeks.	
			- standing EO/ EC;		
			- cyclic L/R head rotation;		
			- cyclic arm lifting and lowering task;		
			- cyclic L/R trunk rotations;		
			- cyclic forward trunk bending.		
**Hagedorn & Holm**[32]	Patients from a geriatric falls and balance clinic.	Randomized controlled trial.	1) Maximal isometric muscle force: knee extensor/ flexor muscle and ankle dorsiflexion.	**Both:** Both groups received progressive resistance muscle strength training whit high intensity, training in step machine, cycling (at least 15 min and minimum of 3 km) and ball games (dribbling, rolling and throwing). Patients were also instructed to train endpoints at home when they scored low in a pre-training test.	- Within group analyses showed significant improvement after the intervention period on knee extension for both groups. CG had significant improvement on STS and EG for walking distance (6 MW test).
	N = 35		2) STS (in 30s).	**EG:** Computer Feedback System- Personics.	- Comparisons between groups showed significant change in time standing on a foam with EC in favoring of EG. There were no differences between groups for others outcomes.
	81.3 ± 6.9 yrs (withdraw = 8)			Four games were used and controlled through weight shifts. Games:	
	EG: n = 15		3) Arm curl test (in 30s).	1- Building a tower used lifting a leg.	
	CG: n = 12		4) TUG.	2- Bursting a balloon with alternated movements for normal standing to toe.	
			5) 6 MW.	3- Controlling a tray with a drink by shifting the body position while standing on medium dense foam.	
			6) MCTSIB: firm and foam surface with EO/ EC.	4- Catching fruits in a bucket.	
			7) OLS.	Two games were allowed at each training session if they did not exceed about 10 minutes. As patients progressed, the surface was changed to a more difficult one.	
			8) Tandem test.	Individual session 2/week (1.5 hour each session) for 12 weeks.	
			9) BBS.	**CG:** Traditional balance training.	
			10) DGI.	Treatment was composed of exercises standing on different surfaces (foam, tilting platforms and pillows) EO/EC, one leg balance training, walking on a line and passing an obstacle course.	
			11) FES-I.	Individual session 2/week (1.5 hour each session) for 12 weeks.	

6 MW = 6 minute walk test; ABC Scale = The Activities-specific Balance Confidence *Scale; AST = Alternate Step Test;* A-P = Antero – Posterior; BBS = Berg Balance Scale; CG = Control Group; COP = center of pressure; CSRT = Choice Stepping Reaction Time; EC = Eyes Closed; EO = Eyes Opened; EG = Experimental Group; FES-I = Falls Efficacy Scale International; FRT = *Functional Reach Test;* DGI = Dynamic Gait Index; GDS = Geriatric Depression Scale; INHIB = Inhibitory Component; L/R = Left/Right; LOS = Limit of Stability; *MCTSIB = Modified Clinical Test of Sensory Interaction and Balance;* M-L = Medio- Lateral; OLS = One Leg Stance; PPA = Physiological Profile Assessment; SAFFE = The Survey of Activities and Fear of Falling in the Elderly; STS = Sit to Stand; TUG = Timed Up and Go; TMT = Trail Making Test; VRE = Virtual Reality Environment.

### Sample characteristics

The samples composition of the 13 studies varied from 12 to 58 participants divided between control group and intervention group. The samples included both sexes, with a higher proportion of women [6–12, 28–32]. Just one of the studies failed to report the proportion of sexes in the sample [13]. Five studies presented a sample size calculation [7, 10, 12, 13, 29] and among these just one had its data analyzed with the initially calculated sample size [29].

Four studies were conducted with community-dwelling elders [9, 13, 28, 30], four with older people from a housing facility/ senior living community [6, 7, 10, 31], three studies took their samples from outpatient clinics (falls and balance clinic/ orthopedic and balance) [8, 12, 32], one study recruited inpatients from a geriatric hospital rehabilitation unit [29], and other older adults attending day hospital [11]. Despite being in different therapeutic settings, the inclusion criteria were similar in all the studies analyzed, demonstrating a relative degree of functional independence and cognitive capacity in the participants. The survey conducted by Laver et al. [29] followed the same criteria for inclusion as the other studies, however, the sample is different because the participants were hospitalized due to acute conditions or health complications such as falls, fractures, medical and surgical problems.

### Assessed outcomes

All studies presented some type of measurement related to the primary outcome of this review, be it through the analysis of mobility [6–11, 13, 29, 32], body balance [9–11, 13, 28, 29, 31, 32] or questionnaires on self-efficacy in activities involving risk of falls [7, 8, 11–13, 29, 30, 32]. For the mobility outcome, the most frequently used instrument was the Timed Up and Go test (TUG) [7, 9–11, 13, 29, 32], while for body balance it was the Berg Balance Scale (BBS) [9, 11, 28, 29, 32] and for self-efficacy it was the Falls Efficacy Scale (FES/ FES-I) [7, 13, 30, 32]. Other tests and instruments related to the primary outcome, were Sit to Stand 5 (STS) or Chair Stand Test [6, 7, 13, 32], The Activities-specific Balance Confidence Scale (ABC scale) [8, 11, 29], Tinetti Performance Oriented Mobility Assessment (POMA) [10, 28, 31], One Leg Stance (OLS) [10, 31, 32], Functional Reach Test (FR) [9, 10], The Survey of Activities and Fear of Short Physical Battery (SPPB) [29], Dynamic Gait Index (DGI) [32], Physiological Profile Assessment (PPA) [13] and Falling in the Elderly (SAFFE) [12].

With regard to the secondary outcomes of this review, the most frequently assessed measures were muscle strength [7, 12, 32]; gait [11, 12, 30], postural control [7, 10, 12, 31] and other parameters [13, 30] through the use of laboratory equipment. Outcomes assessed with lesser degree of representation in the studies were questionnaires on daily living activities [29], quality of life [28, 29], cognition [6, 13], depression [8, 12], aerobic capacity [32] and history of falls.[10] In some studies, the participants in the exergames group were asked about discomfort and adverse events [29], impressions of the game [6], enjoyment [28] and motivation [7].

### Study design and methodological quality

Ten studies described themselves as Randomized Controlled Trials [6–9, 11, 12, 28, 30–32] and three studies as Randomized Controlled Trial – Pilot Studies [10, 13, 29]. Just one study performed analysis of the short-term follow-up (one month after the intervention period) [6]. In the study by Duque et al. [12] during the follow-up, the participants in the exergame group continued to receive their usual care. Thus, no long-term follow-up was performed in any of the studies.

All studies included in this review had their PEDro score retrieved from the PEDro website, since they had been ranked previously (Table 3). Studies scores show that most of the studies presented methodological problems, with a high proportion of scores below 5 [6, 9, 10, 12, 28, 30–32].

**Table 3:** **Methodological analysis of exergames in older adults by the PEDro sclale**

Study	Eligibility criteria	Random allocation	Concealed allocation	Baseline comparability	Blind subjects	Blind therapists	Blind assessors	Adequate follow-up	Intention-to-treat analysis	Between-group comparisons	Point estimates and variability	Score
**Bieryla & Dold**[9]	yes	yes	no	yes	no	no	no	yes	no	yes	yes	5
**Duque et al.**[12]	yes	yes	no	yes	no	no	yes	no	no	yes	yes	5
**Schoene et al.**[13]	yes	yes	yes	yes	no	no	yes	yes	no	yes	yes	7
**Franco et al.**[28]	yes	yes	no	yes	no	no	no	yes	no	yes	yes	5
**Jorgensen et al.**[7]	yes	yes	no	yes	no	no	yes	yes	yes	yes	yes	7
**Laver et al.**[29]	yes	yes	yes	yes	no	no	yes	yes	yes	yes	yes	8
**Maillot & Perrot**[6]	yes	yes	no	yes	no	no	no	yes	no	yes	yes	5
**Pichierri et al.**[30]	yes	yes	no	yes	no	no	no	no	no	yes	yes	4
**Pluchino et al.**[10]	yes	yes	yes	yes	no	no	no	no	no	yes	yes	5
**Rendon et al.**[8]	yes	yes	no	yes	no	no	yes	no	yes	yes	yes	6
**Toulotte et al.**[31]	yes	yes	yes	yes	no	no	no	yes	no	no	yes	5
**Szturm et al.**[11]	yes	yes	yes	yes	no	no	yes	yes	no	yes	yes	7
**Hagedorn & Holm**[32]	yes	yes	no	yes	no	no	no	no	no	yes	yes	4

### Intervention protocol

The exergames groups used games through Nintendo Wii gaming console [6–10, 28, 29, 31] (Table 1), games created on computers [11, 32], Dance video game with pad [13, 30] and the Balance Rehabilitation Unit (BRU™) [12] (Table 2).

Some studies added to the exergames group recommendations for falls prevention [12], warm-up and cool-down exercises [10], adapted physical activity [31], home exercises [28], exercises for balance, muscle resistance and strengthening [30, 32].With the exception of one study that administered the games in pairs [6], the other studies performed the exergames individually. Pichierri et al. [30] combined the exergames performed individually with a group intervention using conventional exercises. In only one study, the participants were instructed to use the exergames at home, with no professional supervision during the sessions [13].

In six studies there was a control group with no type of intervention [6, 8, 9, 13, 28, 31]. The study by Jorgensen et al. [7] had a placebo control group, using EVA insoles with no therapeutic effect. When the control group was active, the therapies performed involved physical activity, conventional physiotherapy, traditional balance training and usual care [10–12, 29–32]. Also with regard to the control group, specific protocols and techniques were used, such as Matter of Balance (MOB) [28] and Tai Chi [10]. Three studies delivered group interventions as control [10, 28, 30].

The time of exergames intervention varied from 10 minutes to an hour, with a predominance of therapies with an average time of 30 minutes. For the studies involving some type of intervention in the control group, the time varied from 25 minutes to an hour. The total duration of treatment varied from 3 to 20 weeks and the frequency of the sessions was from 1 to 5 times a week.

### Effects of the intervention

For the studies that compared exergames and active control, no difference was observed between groups in two of the studies [10, 28]. The other studies demonstrated improvements favoring the exergame group in some of the outcomes assessed, such as laboratory outcomes related to gait and posture control [12, 30, 31], balance [11, 29], mobility [29] and self-efficacy for falls [11, 12].

In the study with the placebo control (EVA insole), there was improvement in the parameters relating to muscle strength, mobility and self-efficacy for falls [7]. The studies that used comparison with non-active control group observed differences only in some specific parameters related to balance [8, 9], mobility [6, 8, 13], muscle strength [6], reaction time [13] and physiological functions involved in postural stability [13] in favor of the exergame group. In one study with three arms: a group using Wii Fit games, an MOB intervention group and a non-active control group it was observed a balance improvement in all groups with no difference between them [28].

For the TUG, the instrument most used by the studies included in this review, three studies indicated improvement in favor of the exergame group [7, 13, 29], one study did not observed a superiority in favor of the exergame group [11] and the others studies did not observed any improvement in any of the groups [9, 10, 32]. With regard to the BBS, three studies obtained significant improvement from interactive game group in comparison with the control group [9, 11, 29], being that Bieryla & Dold [9] observed this improvement only in the follow-up analysis after one month. In the study by Franco et al. [28] there was an increase in the BBS score for the three groups analyzed, but with no significant difference between them. There was no improvement in the results for the BBS in older outpatients from the geriatric and balance clinic [32].

The older adults responded positively in terms of enjoyment and their perception of improvement in functional aspects after practicing the exergames [6, 13, 28]. Four articles demonstrated high levels of adherence to the treatments [6, 12, 13, 30]. Two studies reported that there were no adverse effects during intervention with the use of games [7, 13] while Laver et al. [29] reported cases of discomfort for the exergames group and for the control group that used conventional therapy.

## Discussion

The use of technologies that allow the creation of interactive environments and that provide a form of treatment for the older population by way of VR is spreading worldwide. However, as shown in this review, there is still no substantial evidence of improvement in physical functioning among older adults through the use of exergames, either as a complementary form or as an alternative to other types of intervention.

This systematic review included studies that used different VR technologies to administer the therapies with interactive games. The Nintendo™ Wii (Nintendo; Redmond, WA, USA) console was the one most frequently used to apply the games [6–10, 28, 29, 31] with variations depending on the package and accessories used such as the Wii Sports package with the associated handheld remotes and the balance, fitness and strength games in the Wii Fit package with the accompanying Wii balance board. Some studies used more sophisticated VR systems, such as the computer feedback system (Personics, ÄbyhØj Denmark) coupled to sensors that register body position [32], BRU™ a force platform used to assess (posturography) and train balance [12], and computer games coupled to the flexible pressure mat (FSA pressure mat, Vista Medical td, Winnipeg.) developed exclusively for the study [11]. Another VR option was the use of step pads with the modified Dance Dance Revolution (DDR) Stepmania game [13, 30].

With the exception of BRU, which requires specific training for implementation, has a high cost and depends on ample space and safety accessories, even those that make use of specific computer programs represent a relatively simple form of intervention with virtual games. It is notable that the studies give priority to the games available on commercial consoles that facilitate their use in therapeutic environments and participant access to the equipment if they wish to continue the activities after the study.

According to the World Health Organization [33], the regular practice of physical activities among older adults can prevent against diseases and disability, including tasks of transportation (e.g. walking or cycling), occupational, household chores, play, games, sports or planned exercise, in the context of daily, family and community activities. The use of interactive games through VR can be, therefore, considered as a form of physical activity and this way should obey recommendations related to period and length of practice, to bring the desired benefits to the health of the older person.

The directives of the Global Recommendations on Physical Activity for Health [33] recommend that older people practice at least 150 minutes of moderately aerobic physical activity or at least 75 minutes of vigorously aerobic physical activity over the course of a week. Also according to this guideline, the elderly should carry out activities for muscular strengthening involving large major muscle groups twice a week or more. In the case of older individuals with reduced mobility, an exercise program should be followed three times a week, aimed at improving balance and preventing falls. However, in the case of interactive games there are still no references with regard to the time of intervention needed for functional improvement.

In the studies analyzed in this review, the interventions demanded movements in an orthostatic position which we considered to represent moderately intense physical activity. This being so, none of the studies attained the minimum length of practice time recommended for the older population. This data corroborates the findings of other systematic reviews on VR subject, testifying the lack of standardization in dosage for this type of intervention for therapeutic purposes [21, 34].

The VR games were administered on an isolated basis [6–9, 11, 13, 29] or combined with conventional physical activities [10, 12, 28, 30–32]. Concepts related to learning and motor control consider that practice involving functional tasks that are identifiable through variable practices and with the presence of contextual interferences ensure greater transfer of the task practiced to new tasks and new environments [35]. Interventions that bring together several components such as strength training, resistance, balance exercises, cognitive elements and education stimulate the older person on a broader level and prevent disability [36]. Ultimately, the use of exergames associated with multiple components and variable practice may result in improved functionality and greater gains in the process of rehabilitation compared with practices conducted on a single basis.

With regard to the assessment instruments, a large part of the studies included in this review chose methods that are widely used in the older population, such as BBS, TUG, FR and POMA. These instruments can be applied in a range of therapeutic environments; they do not require complex materials, extended lengths of time for administration and they are low-cost. These characteristics facilitate their use in clinical practice. Nevertheless, some of these instruments might have problems with responsiveness. This fact may be caused, in some of the studies by a ceiling effect on the scores in the baseline assessment, justifying the lack of significant difference between the game intervention group and the control group [9, 28].

As presented in the results, the studies included in this review registered specific improvements in some measures relating to physical functioning. Studies that use assessment based on posturography and gait analysis detected improvements in quantitative laboratorial parameters [12, 30, 31]. These parameters are more sensitive to change, but these gains are not always transferred to the activities assessed in the functional capacity tests. Also, little is known about the differences that are clinically relevant for these instruments. With regard to the instruments, of the seven studies analyzed that had their outcomes assessed by the TUG [7, 9–11, 13, 29, 32], just two of them revealed significant improvement in the group that had intervention of exergames in relation to the control group [7, 29]. In the study by Laver et al. [29] the group that had intervention with Nintendo Wii obtained better results than the traditional hospital therapy group after adjustments to hospital length of stay, age, sex and baseline functional independence measure. Jorgensen et al. [7] compared intervention through Nintendo Wii Training with a placebo. It is worth pointing out that for those who encountered no significant differences between the groups post-intervention, or the test result was within the normal range prior to intervention [9, 10, 13], or the groups presented differences in the baseline [11, 32]. With regard to the BBS, just one study encountered an improvement in favor of the exergame group in comparison with the control group [11]. Franco et al. [28], observed improvement both in the intervention group and in the control group, without any significant difference between them. In the other studies there was no significant difference in the BBS scores between exergame and the control group in the post-intervention period [9, 32]. However, one point that appears to be a consensus is the motivation that the use of VR through interactive games provides [6, 13, 28]. Because it involves tasks that combine physical and cognitive demands in an attractive and challenging way, interactive games may represent an option that is both viable and well-accepted by older people, thereby expanding the therapeutic strategies used in conventional physiotherapy.

Although this review has only chosen randomized clinical trials, aiming to attain better scientific evidence, the studies selected present methodological limitations, and consequently restricting their capacity for generating evidence regarding the efficacy of intervention with VR games. The most frequent limitation is related to the sample size and its characteristics. In order to render the use of interactive games viable, inclusion criteria were applied to ensure a relative degree of physical and cognitive independence of the older adults. This not only makes it more difficult to recruit such participants but also limits the representativeness of the sample, portraying a very specific parcel of the older population within the chosen therapeutic scenarios. The absence of sample size calculation may culminate in systematic errors [37, 38] and the small sample size along with the restrictions in the inclusion criteria reduce the external validity of the study, i.e. that the results of the surveys studies are limited for population generalization. Only three studies performed the intention-to-treat analysis [7, 8, 29] which protects the randomization and preserves compatibility between groups, thereby reducing the chance of bias [39]. Also, the studies used in this review cannot categorically state that the gains observed would lead to lasting benefits for the participants, since no follow-up analysis was performed.

These limitations have been quantified and are expressed by low scores on the PEDro scale. Although the criteria for eligibility, random allocation, baseline comparability, point estimates and variability have been adopted; the absence of intention-to-treat analysis, blinding and follow-up in a large part of the studies result in low scores and indicate poor methodological quality. The other reviews published on VR come up against similar problems [17, 21, 23, 34].

## Conclusion

This manuscript brings a critic view of studies involving the use of exercises using interactive games (exergames) in improving physical functioning in older. Resources involving VR are growing in interest and the use of exergames has shown the potential to represent an option that is both viable and well-accepted by older people. However the results discussed in this review show that evidence to support the effectiveness of using exergames for improving physical functioning in older adults remains inconclusive.

In order to achieve better methodological quality and provide stronger scientific evidence, it is necessary that future studies use more responsive instruments with clear minimal clinically important difference, as well as conduct appropriate statistical analyses and sample size calculations. We observed that there was a wide variability in exercise prescription regarding the use of exergames, ultimately making a suitable dosage of training still inconceivable. It is recommended that RCTs should follow the Consolidated Standards of Reporting Trials [40], since it was challenging to retrieve relevant information in some studies. Therefore, it is still not possible to recommend the use of exergames as an isolated or combined intervention for improving physical functioning in older people.

## Authors’ original submitted files for images

Below are the links to the authors’ original submitted files for images. Authors’ original file for figure 1
