# Supplementary figures and images for: Virtual reality using games for improving physical functioning in older adults: a systematic review
Source: J Neuroeng Rehabil. 2014 Nov 15;11:156. doi: 10.1186/1743-0003-11-156 (PMC4247561; doi:10.1186/1743-0003-11-156)

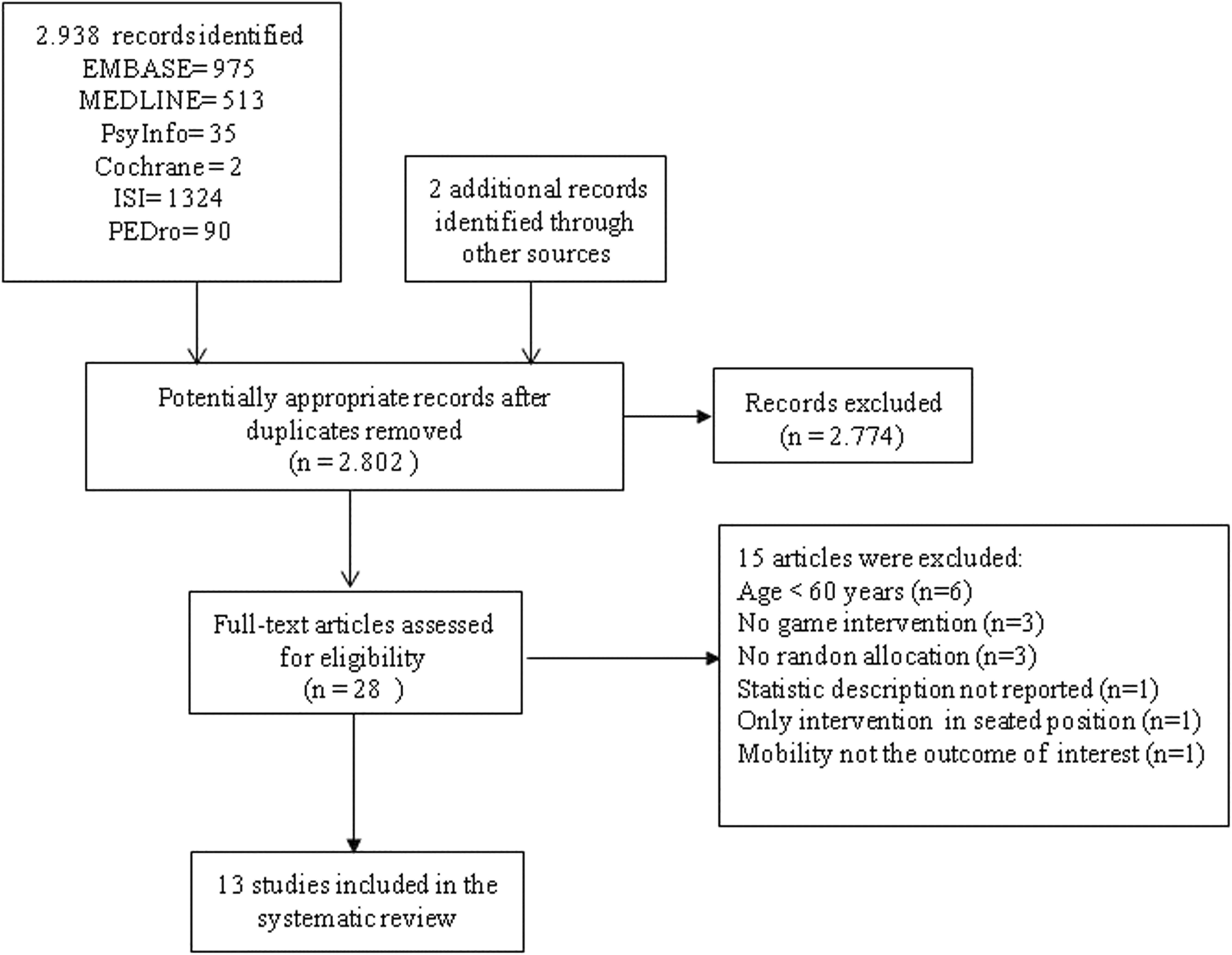

Supplement: Supplementary file 1 — Authors’ original file for figure 1 [file 12984_2014_675_MOESM1_ESM.tif]
